# Supplementary material for: Charge-4e superconductivity and chiral metal in 45°-twisted bilayer cuprates and related bilayers
Source: Nat Commun. 2023 Dec 1;14:7926. doi: 10.1038/s41467-023-43782-2 (PMC10692084; doi:10.1038/s41467-023-43782-2)
Supplement: Supplementary file 1 — Supplementary Information for [file 41467_2023_43782_MOESM1_ESM.pdf]

Supplementary Information for " Charge-4e superconductivity and chiral metal in the $45^{\circ}-$ twisted bilayer cuperates and related bilayers "

Yu-Bo Liu,[1,*] Jing Zhou,[2,3,*] Congjun Wu,[4,5,6,7] and Fan Yang[1,†]

[1] *School of Physics, Beijing Institute of Technology, Beijing 100081, China*
[2] *Department of Science, Chongqing University of Posts and Telecommunications, Chongqing 400065, China*
[3] *Institute for Advanced Sciences, Chongqing University of Posts and Telecommunications, Chongqing, 400065, China*
[4] *Institute for Theoretical Sciences, WestLake University, 310024, Hangzhou, China*
[5] *New Cornerstone Science Laboratory, Department of Physics, School of Science, Westlake University, 310024, Hangzhou, China*
[6] *Key Laboratory for Quantum Materials of Zhejiang Province, Department of Physics, School of Science, Westlake University, Hangzhou 310030, P. R. China*
[7] *Institute of Natural Sciences, Westlake Institute for Advanced Study, Hangzhou 310024, P. R. China*

(Dated: October 30, 2023)

* These two authors contributed equally to this work.
† yangfan_blg@bit.edu.cn

Inventory of Supplementary Information:

Sec. I: Derivation of the effective Hamiltonian from Ginzburg-Landau theory.

Sec. II: Derivation of the effective Hamiltonian of chiral TSC in monolayers systems from G-L theory.

Sec. III: Stability analysis of the fixed points.

Sec. IV: More detailed Results about the RG study.

Sec. V: More details Results about the MC study.

Sec. VI: The MC result without considering kinematic constraint(γ=0).

I. DERIVATION OF THE EFFECTIVE HAMILTONIAN FROM GINZBURG-LANDAU THEORY

In this section, we derive the effective Hamiltonian appearing in the Eq. (12) of the main text by expanding the Ginzburg-Landau (G-L) free energy up to the fourth-order term of the order parameters.

A. Symmetry

To elucidate the effect of the symmetry operations on the argument of the G-L free-energy functional, let's start from the mean-field BCS Hamiltonian:

$$H_{\text{BCS-MF}} = H_{\text{TB}} + \sum_{\mathbf{r},\delta} c^{\dagger}_{\mathbf{r},\text{t}\uparrow} c^{\dagger}_{\mathbf{r}+\delta,\text{t}\downarrow} \Gamma^{(\text{t})}(\delta)\psi_{\text{t}}(\mathbf{r}) + c^{\dagger}_{\mathbf{r},\text{b}\uparrow} c^{\dagger}_{\mathbf{r}+\delta,\text{b}\downarrow} \Gamma^{(\text{b})}(\delta)\psi_{\text{b}}(\mathbf{r}) + h.c. \tag{S1}$$

Here $\mathbf{r}$ labels the center-of-mass coordinate of a Cooper pair and δ is the relative coordinate between the two electrons within a Cooper pair. $\Gamma^{(\mu)}(\delta)$ is the fixed normalized real form factor with μ = t/b, and $\psi_\mu(\mathbf{r})$ is a slowly-varying "envelope" function describing the spatial fluctuation of the complex pairing amplitude at finite temperature. Each symmetry operation first acts on the c and $c^\dagger$ operators, then through a dummy-index transformation, the effect is transferred to the action of the Γ and ψ. As the Γ has simple transformation rule under the symmetry, i.e. it changes sign upon every C_n^1 operation and changes or does not change upon the mirror reflection operation, the effect can be transferred purely to ψ. Therefore, we have chosen an gauge in which each symmetry operation only acts on $\psi_\mu(\mathbf{r})$.

Under $\widetilde{C}_{2n}^1$, the spatial dependent pairing amplitudes change to:

$$\psi_{\text{b}}(\mathbf{r}) \to \widetilde{\psi}_{\text{b}}(\mathbf{r}) = \psi_{\text{t}}(\widehat{P}_{\frac{\pi}{n}}^{-1}\mathbf{r}), \qquad \psi_{\text{t}}(\mathbf{r}) \to \widetilde{\psi}_{\text{t}}(\mathbf{r}) = -\psi_{\text{b}}(\widehat{P}_{\frac{\pi}{n}}^{-1}\mathbf{r}). \tag{S2}$$

Under the mirror reflection operation $\widehat{P}$, it is easy to prove (we have chosen a gauge without loss of generality):

$$\psi_{\text{b}}(\mathbf{r}) \to \widetilde{\psi}_{\text{b}}(\mathbf{r}) = -\psi_{\text{b}}(\widehat{P}^{-1}\mathbf{r}), \qquad \psi_{\text{t}}(\mathbf{r}) \to \widetilde{\psi}_{\text{t}}(\mathbf{r}) = \psi_{\text{t}}(\widehat{P}^{-1}\mathbf{r}). \tag{S3}$$

For convenience, we rotate the basis to $\psi_\pm = \psi_t \pm i\psi_b$ and rewrite the above transformation in the $\mathbf{k}-$space

$$\begin{aligned}
\psi_+(\mathbf{k}) &\xrightarrow{\widetilde{C}^1_{2n}} e^{i\pi/2}\psi_+(\widehat{P}^{-1}_{\frac{\pi}{n}}\mathbf{k}), \qquad & \psi_-(\mathbf{k}) &\xrightarrow{\widetilde{C}^1_{2n}} e^{-i\pi/2}\psi_-(\widehat{P}^{-1}_{\frac{\pi}{n}}\mathbf{k}) \\
\psi_+(\mathbf{k}) &\xrightarrow{\widehat{P}} \psi_-(\widehat{P}^{-1}\mathbf{k}), & \psi_-(\mathbf{k}) &\xrightarrow{\widehat{P}} \psi_+(\widehat{P}^{-1}\mathbf{k}).
\end{aligned} \tag{S4}$$

Here we consider the $\widetilde{C}^1_{2n}$ and the mirror reflection, but neglect the time-reversal symmetry. The final effect of the time-reversal symmetry on the Hamiltonian is consistent with that obtained with only considering the $\widetilde{C}^1_{2n}$ and the mirror reflection symmetries.

With the definition $\mathbf{k}_\pm = k_x \pm ik_y$, we obtain the momentum transformation relations:

$$\widehat{P}_{\frac{\pi}{n}}\mathbf{k}_+ = e^{i\pi/n}\mathbf{k}_+, \qquad \widehat{P}_{\frac{\pi}{n}}\mathbf{k}_- = e^{-i\pi/n}\mathbf{k}_-. \tag{S5}$$

B. The second-order G-L expansion

Up to the lowest-order expansion, the differential term in G-L free energy has the following general form in the $\mathbf{k}-$space:

$$\begin{aligned}
F_0^{(2)} &= \sum_{\mathbf{k}} \psi_+^*(\mathbf{k})\psi_+(\mathbf{k})(a_1\mathbf{k}_+^2 + b_1\mathbf{k}_-^2 + c_1\mathbf{k}_+\mathbf{k}_-) \\
&+ \sum_{\mathbf{k}} \psi_+^*(\mathbf{k})\psi_-(\mathbf{k})(a_2\mathbf{k}_+^2 + b_2\mathbf{k}_-^2 + c_2\mathbf{k}_+\mathbf{k}_-) \\
&+ \sum_{\mathbf{k}} \psi_-^*(\mathbf{k})\psi_+(\mathbf{k})(a_3\mathbf{k}_+^2 + b_3\mathbf{k}_-^2 + c_3\mathbf{k}_+\mathbf{k}_-) \\
&+ \sum_{\mathbf{k}} \psi_-^*(\mathbf{k})\psi_-(\mathbf{k})(a_4\mathbf{k}_+^2 + b_4\mathbf{k}_-^2 + c_4\mathbf{k}_+\mathbf{k}_-).
\end{aligned} \tag{S6}$$

Under the operation $\widetilde{C}^1_{2n}$, $F_0^{(2)}$ change to:

$$\begin{aligned}
F_0^{(2)} \xrightarrow{\widetilde{C}^1_{2n}} &= \sum_{\mathbf{k}} \psi_+^*(\mathbf{k})\psi_+(\mathbf{k})(a_1e^{i2\pi/n}\mathbf{k}_+^2 + b_1e^{-i2\pi/n}\mathbf{k}_-^2 + c_1\mathbf{k}_+\mathbf{k}_-) \\
&+ \sum_{\mathbf{k}} e^{-i2\pi/2}\psi_+^*(\mathbf{k})\psi_-(\mathbf{k})(a_2e^{i2\pi/n}\mathbf{k}_+^2 + b_2e^{-i2\pi/n}\mathbf{k}_-^2 + c_2\mathbf{k}_+\mathbf{k}_-) \\
&+ \sum_{\mathbf{k}} e^{i2\pi/2}\psi_-^*(\mathbf{k})\psi_+(\mathbf{k})(a_3e^{i2\pi/n}\mathbf{k}_+^2 + b_3e^{-i2\pi/n}\mathbf{k}_-^2 + c_3\mathbf{k}_+\mathbf{k}_-) \\
&+ \sum_{\mathbf{k}} \psi_-^*(\mathbf{k})\psi_-(\mathbf{k})(a_4e^{i2\pi/n}\mathbf{k}_+^2 + b_4e^{-i2\pi/n}\mathbf{k}_-^2 + c_4\mathbf{k}_+\mathbf{k}_-).
\end{aligned} \tag{S7}$$

As $n = 4$ or 6, the invariance of $F_0^{(2)}$ requires only $c_1, c_4 \neq 0$ while all the other coefficients keep zero. Further more, $c_1 = c_4 = B$ is required by the mirror-reflection symmetry. Changing back to the real space, we arrive at the form of $F_0^{(2)}$ as following:

$$\begin{aligned}
F_0^{(2)} &= B\int d^2\mathbf{r}[(\nabla\psi_+^*)\cdot(\nabla\psi_+) + (\nabla\psi_-^*)\cdot(\nabla\psi_-)] \\
&= B\psi_0^2\int d^2\mathbf{r}[\nabla(e^{-i\theta_t} - ie^{-i\theta_b})\cdot\nabla(e^{i\theta_t} + ie^{i\theta_b}) + \nabla(e^{-i\theta_t} + ie^{-i\theta_b})\cdot\nabla(e^{i\theta_t} - ie^{i\theta_b})] \\
&= 2B\psi_0^2\int d^2\mathbf{r}[(\nabla\theta_b)^2 + (\nabla\theta_t)^2] \\
&= 4B\psi_0^2\int d^2\mathbf{r}[|\nabla\theta_+|^2 + |\nabla\theta_-|^2]
\end{aligned} \tag{S8}$$

Here ψ_0 represents the amplitude of the pairing order parameter.

C. The fourth-order G-L expansion

According to the second order expansion of the differential term in the G-L free energy, the coefficients before θ_+ and θ_- are the same. To get different coefficients, we need expand F_0 to the fourth order with the general form as:

$$F_0^{(4)} = \sum_{\mathbf{k}_1,\mathbf{k}_2,\mathbf{k}_3,\mathbf{k}_4} \psi^*_\alpha(\mathbf{k}_1)\psi^*_\beta(\mathbf{k}_2)\psi_\gamma(\mathbf{k}_3)\psi_\nu(\mathbf{k}_4)(\sum_{i,j=1}^{4} \alpha_{ij}\mathbf{k}_{+i}\cdot\mathbf{k}_{+j} + \beta_{ij}\mathbf{k}_{-i}\cdot\mathbf{k}_{-j} + \gamma_{ij}\mathbf{k}_{+i}\cdot\mathbf{k}_{-j} + \nu_{ij}\mathbf{k}_{-i}\cdot\mathbf{k}_{+j}) \tag{S9}$$

where $\alpha, \beta, \gamma, \nu = \pm$. It is easy to verify that $\alpha+\beta+\gamma+\nu$ should be an even integer. Since the angular momentum of $\psi_\alpha^{(*)}(\mathbf{k}_i)$ is $\pm n/2$, that of $\psi^*_\alpha(\mathbf{k}_1)\psi^*_\beta(\mathbf{k}_2)\psi_\gamma(\mathbf{k}_3)\psi_\nu(\mathbf{k}_4)$ should be an integer times n. And the angular momentum of $k_\pm$ is ±1. The invariance of $F_0^{(4)}$ under $\widetilde{C}_{2n}^1$ requires that the total angular momentum should be zero. For $n=4$ or 6, the restriction of zero total angular momentum dictates $\alpha_{ij} = \beta_{ij} = 0$. Then, we can simplify the general form of $F_0^{(4)}$:

$$\begin{aligned} F_0^{(4)} &= \sum_{\mathbf{k}_1,\mathbf{k}_2,\mathbf{k}_3,\mathbf{k}_4} \psi^*_+(\mathbf{k}_1)\psi^*_+(\mathbf{k}_2)\psi_+(\mathbf{k}_3)\psi_+(\mathbf{k}_4)(\sum_{i,j=1}^{4} 2\gamma_{ij}^{(1)}\mathbf{k}_i\cdot\mathbf{k}_j) \\ &+ \sum_{\mathbf{k}_1,\mathbf{k}_2,\mathbf{k}_3,\mathbf{k}_4} \psi^*_+(\mathbf{k}_1)\psi^*_-(\mathbf{k}_2)\psi_+(\mathbf{k}_3)\psi_-(\mathbf{k}_4)(\sum_{i,j=1}^{4} 2\gamma_{ij}^{(2)}\mathbf{k}_i\cdot\mathbf{k}_j) \\ &+ \sum_{\mathbf{k}_1,\mathbf{k}_2,\mathbf{k}_3,\mathbf{k}_4} \psi^*_-(\mathbf{k}_1)\psi^*_-(\mathbf{k}_2)\psi_-(\mathbf{k}_3)\psi_-(\mathbf{k}_4)(\sum_{i,j=1}^{4} 2\gamma_{ij}^{(3)}\mathbf{k}_i\cdot\mathbf{k}_j) \end{aligned} \tag{S10}$$

We now consider the first and the third term in the general form of $F_0^{(4)}$ since there is only ψ_+ or ψ_-. It is easy to verify that $\psi_\pm \to \psi^*_\mp$ under TRS. Remembering all the transformation relation in mind, the form of equation (S10) can be further simplified as:

$$\begin{aligned} F_{0(1,3)}^{(4)} = \sum_{\mathbf{k}_1,\mathbf{k}_2,\mathbf{k}_3,\mathbf{k}_4} &[\psi^*_+(\mathbf{k}_1)\psi^*_+(\mathbf{k}_2)\psi_+(\mathbf{k}_3)\psi_+(\mathbf{k}_4) + \psi^*_-(\mathbf{k}_1)\psi^*_-(\mathbf{k}_2)\psi_-(\mathbf{k}_3)\psi_-(\mathbf{k}_4)] \\ &\cdot[a(\mathbf{k}_1^2+\mathbf{k}_2^2+\mathbf{k}_3^2+\mathbf{k}_4^2) + b(\mathbf{k}_1\cdot\mathbf{k}_2+\mathbf{k}_3\cdot\mathbf{k}_4) + c(\mathbf{k}_1+\mathbf{k}_2)\cdot(\mathbf{k}_3+\mathbf{k}_4)] \end{aligned} \tag{S11}$$

A valuable equation $(\mathbf{k}_1+\mathbf{k}_2-\mathbf{k}_3-\mathbf{k}_4)^2 = 0$ should be emphasized before the proceeding process. Expanding this equation, we have:

$$\sum_{i=1}^{4}\mathbf{k}_i^2 = 2(\mathbf{k}_1+\mathbf{k}_2)\cdot(\mathbf{k}_3+\mathbf{k}_4) - 2(\mathbf{k}_1\cdot\mathbf{k}_2+\mathbf{k}_3\cdot\mathbf{k}_4). \tag{S12}$$

We can rewrite the first and third term:

$$\begin{aligned} F_{0(1,3)}^{(4)} = \sum_{\mathbf{k}_1,\mathbf{k}_2,\mathbf{k}_3,\mathbf{k}_4} &[\psi^*_+(\mathbf{k}_1)\psi^*_+(\mathbf{k}_2)\psi_+(\mathbf{k}_3)\psi_+(\mathbf{k}_4) + \psi^*_-(\mathbf{k}_1)\psi^*_-(\mathbf{k}_2)\psi_-(\mathbf{k}_3)\psi_-(\mathbf{k}_4)] \\ &\cdot[(b-2a)\cdot(\mathbf{k}_1\cdot\mathbf{k}_2+\mathbf{k}_3\cdot\mathbf{k}_4) + (c+2a)\cdot(\mathbf{k}_1+\mathbf{k}_2)\cdot(\mathbf{k}_3+\mathbf{k}_4)] \end{aligned} \tag{S13}$$

By the same method, the second term of the fourth order expansion of the differential term in G-L free energy is

$$\begin{aligned} F_{0(2)}^{(4)} &= \sum_{\mathbf{k}_1,\mathbf{k}_2,\mathbf{k}_3,\mathbf{k}_4} \psi^*_+(\mathbf{k}_1)\psi^*_-(\mathbf{k}_2)\psi_+(\mathbf{k}_3)\psi_-(\mathbf{k}_4) \\ &\cdot \quad [a'(\mathbf{k}_1^2+\mathbf{k}_2^2+\mathbf{k}_3^2+\mathbf{k}_4^2) + b'(\mathbf{k}_1\cdot\mathbf{k}_2+\mathbf{k}_3\cdot\mathbf{k}_4) + c'(\mathbf{k}_1\cdot\mathbf{k}_3+\mathbf{k}_2\cdot\mathbf{k}_4) + d'(\mathbf{k}_1\cdot\mathbf{k}_4+\mathbf{k}_2\cdot\mathbf{k}_3)] \end{aligned} \tag{S14}$$

Transforming to the real space, the total form of $F_0^{(4)}$ is:

$$\begin{aligned}
F^{(4)} &= -(b-2a)\int d^2\mathbf{r}[(\nabla\psi_+^*)^2\psi_+^2 + (\psi_+^*)^2(\nabla\psi_+)^2 + (\nabla\psi_-^*)^2\psi_-^2 + (\psi_-^*)^2(\nabla\psi_-)^2] \\
&+ (c+2a)\int d^2\mathbf{r}[\nabla(\psi_+^{*2})\cdot\nabla(\psi_+^2) + \nabla(\psi_-^{*2})\cdot\nabla(\psi_-^2)] \\
&- (b'-2a')\int d^2\mathbf{r}[(\nabla\psi_+^*)\cdot(\nabla\psi_-^*)\psi_+\psi_- + \psi_+^*\psi_-^*(\nabla\psi_+)\cdot(\nabla\psi_-)] \\
&+ (c'+2a')\int d^2\mathbf{r}[\nabla\psi_+^*\cdot\nabla\psi_+|\psi_-|^2 + |\psi_+|^2\nabla\psi_-^*\cdot\nabla\psi_-] \\
&+ (d'+2a')\int d^2\mathbf{r}[\nabla\psi_+^*\cdot\nabla\psi_-\psi_-^*\psi_+ + \nabla\psi_-^*\cdot\nabla\psi_+\psi_+^*\psi_-] \\
&= 32(b+2c+2a)\psi_0^4\int d^2\mathbf{r}|\nabla\theta_+|^2 + 16(c'+2a')\psi_0^4\int d^2\mathbf{r}|\nabla\theta_-|^2. \qquad \text{(S15)}
\end{aligned}$$

So, the stiffness parameters ρ and κ in the text take the form as:

$$\rho = 8B\psi_0^2 + 64(b+c)\psi_0^4, \qquad \text{(S16)}$$

$$\kappa = 8B\psi_0^2 + 32(c'+2a')\psi_0^4. \qquad \text{(S17)}$$

And the the lowest order of the real space Hamiltonian is given by:

$$H_0 = \int d^2\mathbf{r}\left(\frac{\rho}{2}|\nabla\theta_+|^2 + \frac{\kappa}{2}|\nabla\theta_-|^2\right) \qquad \text{(S18)}$$

II. DERIVATION OF THE EFFECTIVE HAMILTONIAN OF CHIRAL TSC IN MONOLAYERS SYSTEMS FROM G-L THEORY

In this section, as the contrast to the previous section, we derive the effective Hamiltonian of chiral TSC in monolayers systems by expanding the G-L free energy up to the second-order term of the order parameters. In the following derivation, we take the $d+id$ TSC in the hexagonal lattice as an example. The final result shows that there are extra dynamic couplings of θ_+ and θ_-.

A. Symmetry

To elucidate the effect of the symmetry operations on the argument of the G-L free-energy functional, let's start from the mean-field BCS Hamiltonian:

$$H_{\text{BCS-MF}} = H_{\text{TB}} + \sum_{\mathbf{r},\delta} c_{\mathbf{r}\uparrow}^\dagger c_{\mathbf{r}+\delta\downarrow}^\dagger \Gamma^{(1)}(\delta)\psi_1(\mathbf{r}) + c_{\mathbf{r}\uparrow}^\dagger c_{\mathbf{r}+\delta\downarrow}^\dagger \Gamma^{(2)}(\delta)\psi_2(\mathbf{r}) + h.c. \qquad \text{(S19)}$$

Here 1 and 2 mark the two degenerate components of the d-wave SC. The interpretation of the Eq. (S19) is the same as the Eq. (S1) except that the component index $1,2$ replaces the layer index t,b.

For convenience, we rotate the basis to $\psi_\pm = \psi_1 \pm i\psi_2$ and rewrite the above transformation in the $\mathbf{k}-$space. Under C_6^1, the spatial dependent pairing amplitudes change to:

$$\psi_+(\mathbf{k}) \to \widetilde{\psi}_+(\mathbf{k}) = e^{i2\pi/3}\psi_+(\widehat{P}_{\frac{\pi}{3}}^{-1}\mathbf{k}), \qquad \psi_-(\mathbf{k}) \to \widetilde{\psi}_-(\mathbf{k}) = e^{-i2\pi/3}\psi_-(\widehat{P}_{\frac{\pi}{3}}^{-1}\mathbf{k}). \qquad \text{(S20)}$$

Under the mirror reflection operation $\widehat{P}$, it is easy to prove (we have chosen a gauge without loss of generality):

$$\psi_+(\mathbf{k}) \to \widetilde{\psi}_+(\mathbf{k}) = \psi_-(\widehat{P}^{-1}\mathbf{k}), \qquad \psi_-(\mathbf{k}) \to \widetilde{\psi}_-(\mathbf{k}) = \psi_+(\widehat{P}^{-1}\mathbf{k}). \qquad \text{(S21)}$$

Here we consider the C_6^1 and the mirror reflection, but neglect the time-reversal symmetry. The final effect of the time-reversal symmetry on the Hamiltonian is consistent with that obtained with only considering the C_6^1 and the mirror reflection symmetries.

With the definition $\mathbf{k}_\pm = k_x \pm ik_y$, we obtain the momentum transformation relations:

$$\widehat{P}_{\frac{\pi}{3}}\mathbf{k}_+ = e^{i\pi/3}\mathbf{k}_+, \qquad \widehat{P}_{\frac{\pi}{3}}\mathbf{k}_- = e^{-i\pi/3}\mathbf{k}_-. \qquad \text{(S22)}$$

B. The second-order G-L expansion

Up to the lowest-order expansion, the differential term in G-L free energy has the following general form in the $\mathbf{k}-$ space:

$$\begin{aligned} F_0^{(2)} &= \sum_{\mathbf{k}} \psi_+^*(\mathbf{k})\psi_+(\mathbf{k})(a_1\mathbf{k}_+^2 + b_1\mathbf{k}_-^2 + c_1\mathbf{k}_+\mathbf{k}_-) \\ &+ \sum_{\mathbf{k}} \psi_+^*(\mathbf{k})\psi_-(\mathbf{k})(a_2\mathbf{k}_+^2 + b_2\mathbf{k}_-^2 + c_2\mathbf{k}_+\mathbf{k}_-) \\ &+ \sum_{\mathbf{k}} \psi_-^*(\mathbf{k})\psi_+(\mathbf{k})(a_3\mathbf{k}_+^2 + b_3\mathbf{k}_-^2 + c_3\mathbf{k}_+\mathbf{k}_-) \\ &+ \sum_{\mathbf{k}} \psi_-^*(\mathbf{k})\psi_-(\mathbf{k})(a_4\mathbf{k}_+^2 + b_4\mathbf{k}_-^2 + c_4\mathbf{k}_+\mathbf{k}_-). \end{aligned} \tag{S23}$$

Under the operation C_6^1, $F_0^{(2)}$ change to:

$$\begin{aligned} F_0^{(2)} \xrightarrow{C_6^1} &= \sum_{\mathbf{k}} \psi_+^*(\mathbf{k})\psi_+(\mathbf{k})(a_1 e^{i2\pi/3}\mathbf{k}_+^2 + b_1 e^{-i2\pi/3}\mathbf{k}_-^2 + c_1\mathbf{k}_+\mathbf{k}_-) \\ &+ \sum_{\mathbf{k}} e^{-i4\pi/3}\psi_+^*(\mathbf{k})\psi_-(\mathbf{k})(a_2 e^{i2\pi/3}\mathbf{k}_+^2 + b_2 e^{-i2\pi/3}\mathbf{k}_-^2 + c_2\mathbf{k}_+\mathbf{k}_-) \\ &+ \sum_{\mathbf{k}} e^{i4\pi/3}\psi_-^*(\mathbf{k})\psi_+(\mathbf{k})(a_3 e^{i2\pi/3}\mathbf{k}_+^2 + b_3 e^{-i2\pi/3}\mathbf{k}_-^2 + c_3\mathbf{k}_+\mathbf{k}_-) \\ &+ \sum_{\mathbf{k}} \psi_-^*(\mathbf{k})\psi_-(\mathbf{k})(a_4 e^{i2\pi/3}\mathbf{k}_+^2 + b_4 e^{-i2\pi/3}\mathbf{k}_-^2 + c_4\mathbf{k}_+\mathbf{k}_-). \end{aligned} \tag{S24}$$

the invariance of $F_0^{(2)}$ requires only $c_1, b_2, a_3, c_4 \neq 0$ while all the other coefficients keep zero. Further more, $c_1 = c_4 = B$ and $b_2 = a_3 = C$ are required by the mirror-reflection symmetry. Changing back to the real space, we arrive at the form of $F_0^{(2)}$ as following:

$$\begin{aligned} F_0^{(2)} &= \int d^2\mathbf{r} B[(\nabla\psi_+^*)\cdot(\nabla\psi_+) + (\nabla\psi_-^*)\cdot(\nabla\psi_-)] \\ &+ C[(\nabla_+\psi_-^*)\cdot(\nabla_+\psi_+) + (\nabla_-\psi_+^*)\cdot(\nabla_-\psi_-)] \end{aligned} \tag{S25}$$

Where the C term is the extra dynamic coupling of θ_+ and θ_-. Similar to the above derivation, such extra dynamic coupling is present in all possible chiral TSC ($p+ip$,$d+id$ in hexagonal lattice and $p+ip$ in square lattice) in monolayers systems.

III. STABILITY ANALYSIS OF THE FIXED POINTS

By the standard RG analysis, the flow equations at the one-loop level are given by:

$$\begin{aligned} \frac{dg_{2,0}}{d\ln b} &= (2-\pi\rho^{'})g_{2,0} \\ \frac{dg_{0,2}}{d\ln b} &= (2-\pi\kappa^{'})g_{0,2} \\ \frac{dg_{1,1}}{d\ln b} &= \left(2-\frac{\pi}{4}(\rho^{'}+\kappa^{'})\right)g_{1,1} \\ \frac{dg_4}{d\ln b} &= (2-\frac{4}{\pi\kappa^{'}})g_4 \\ \frac{d\rho^{'}}{d\ln b} &= -16g_{2,0}^2\rho^{'3} - \frac{g_{1,1}^2}{2}\rho^{'2}(\rho^{'}+\kappa^{'}) \\ \frac{d\kappa^{'}}{d\ln b} &= \frac{256g_4^2}{\pi^4\kappa^{'}} - 16g_{0,2}^2\kappa^{'3} - \frac{g_{1,1}^2}{2}\kappa^{'2}(\rho^{'}+\kappa^{'}), \end{aligned} \tag{S26}$$

Table I. Fixed points of the coupling parameters under RG, and the corresponding phases.

$g_{2,0}$	$g_{0,2}$	g_4	$g_{1,1}$	$\rho^{'}$	$\kappa^{'}$	phase
∞	∞	0	∞	0	0	normal
0	∞	0	0	$> 8/\pi$	0	charge 4e SC
0	0	∞	0	$> 2/\pi$	∞	chiral SC
∞	0	∞	0	0	∞	chiral metal

In the methods of the main text, we have outlined the general process of the stability analysis of the fixed points. In the following, we present more details of the stability analysis for the corresponding four phases shown in Table I.

(i)**The normal phase**:
We define $\bar{g}_{2,0} = \frac{1}{g_{2,0}}$, $\bar{g}_{0,2} = \frac{1}{g_{0,2}}$, $\bar{g}_{11} = \frac{1}{g_{11}}$ and $\ell = \ln b$ to simplify the calculation in the following. The RG flow equation can be rewritten as:

$$\begin{aligned}
\frac{d\bar{g}_{2,0}}{d\ell} &= -(2-\pi\rho^{'})\bar{g}_{2,0} \\
\frac{d\bar{g}_{0,2}}{d\ell} &= -(2-\pi\kappa^{'})\bar{g}_{0,2} \\
\frac{d\bar{g}_{1,1}}{d\ell} &= -\left(2-\frac{\pi}{4}(\rho^{'}+\kappa^{'})\right)\bar{g}_{1,1} \\
\frac{dg_4}{d\ell} &= (2-\frac{4}{\pi\kappa^{'}})g_4 \\
\frac{d\rho^{'}}{d\ell} &= -16\bar{g}_{2,0}^{-2}\rho^{'3} - \frac{1}{2}\bar{g}_{1,1}^{-2}\rho^{'2}(\rho^{'}+\kappa^{'}) \\
\frac{d\kappa^{'}}{d\ell} &= \frac{256g_4^2}{\pi^4\kappa^{'}} - 16\bar{g}_{0,2}^{-2}\kappa^{'3} - \frac{1}{2}\bar{g}_{1,1}^{-2}\kappa^{'2}(\rho^{'}+\kappa^{'}),
\end{aligned} \tag{S27}$$

The M matrix can be obtained as:

$$M = \begin{pmatrix}
-(2-\pi\rho^{'}) & 0 & 0 & 0 & \pi\bar{g}_{2,0} & 0 \\
0 & -(2-\pi\kappa^{'}) & 0 & 0 & 0 & \pi\bar{g}_{0,2} \\
0 & 0 & -\left(2-\frac{\pi}{4}(\rho^{'}+\kappa^{'})\right) & 0 & \frac{\pi}{4}\bar{g}_{1,1} & \frac{\pi}{4}\bar{g}_{1,1} \\
0 & 0 & 0 & 2-\frac{4}{\pi\kappa^{'}} & 0 & \frac{4g_4}{\pi\kappa^{'2}} \\
\frac{32\rho^{'3}}{\bar{g}_{2,0}^3} & 0 & \frac{\rho^{'2}(\rho^{'}+\kappa^{'})}{\bar{g}_{1,1}^3} & 0 & -\frac{48\rho^{'2}}{\bar{g}_{2,0}^2} - \frac{3\rho^{'2}+2\rho^{'}\kappa^{'}}{2\bar{g}_{1,1}^2} & -\frac{\rho^{'2}}{2\bar{g}_{1,1}^2} \\
0 & \frac{32\kappa^{'3}}{\bar{g}_{0,2}^3} & \frac{\kappa^{'2}(\rho^{'}+\kappa^{'})}{\bar{g}_{1,1}^3} & \frac{512g_4}{\pi^4\kappa^{'}} & -\frac{\kappa^{'2}}{2\bar{g}_{1,1}^2} & -\frac{256g_4^2}{\pi^4\kappa^{'2}} - \frac{48\kappa^{'2}}{\bar{g}_{0,2}^2} - \frac{2\kappa^{'}\rho^{'}+3\kappa^{'2}}{2\bar{g}_{1,1}^2}
\end{pmatrix} \tag{S28}$$

Now, let's analyze the order of four coupling parameters at the fixed point. We immediately have $\bar{g}_{0,2} \sim e^{-2\ell}$, $\bar{g}_{2,0} \sim e^{-2\ell}$, $\bar{g}_{1,1} \sim e^{-2\ell}$, and $g_4 \sim e^{-\infty\ell} = 0$. Then, we start to analyze the order of $\rho^{'}$ and $\kappa^{'}$. At the beginning, we can neglect the first term in the RG flow equation of stiffness $\kappa^{'}$ since the order of g_4 is extra low. We should discuss in three cases:
(a)If $O(\rho^{'}) < O(\kappa^{'})$, we can only keep the highest order in the RG equation.

$$\frac{d\kappa^{'}}{d\ell} = -\frac{16\kappa^{'3}}{\bar{g}_{0,2}^2} - \frac{\kappa^{'3}}{2\bar{g}_{1,1}^2} \sim -e^{4\ell}\kappa^{'3} \tag{S29}$$

We immediately get the order of $\kappa^{'}$ as $e^{-2\ell}$. The differential equation of $\rho^{'}$ can be simplified as:

$$\frac{d\rho^{'}}{d\ell} = -\frac{\rho^{'2}\kappa^{'}}{2\bar{g}_{1,1}^2} \tag{S30}$$

The order of $\rho^{'}$ is $e^{-2\ell}$. So, we reach the result $O(\rho^{'}) = O(\kappa^{'}) \sim e^{-2\ell}$, which contradicts the previous assumption.

(b)If $O(\rho^{'}) > O(\kappa^{'})$, the RG flow equation is simplified as:

$$\frac{d\rho^{'}}{d\ell} \sim -e^{4\ell}\rho^{'3};$$
$$\frac{d\kappa^{'}}{d\ell} \sim -\frac{\kappa^{'2}\rho^{'}}{2\bar{g}_{1,1}^2} \tag{S31}$$

Solving the differential equations, we still have $O(\rho^{'}) = O(\kappa^{'}) \sim e^{-2\ell}$ which contradicts the previous assumption.
(c)If $O(\rho^{'}) = O(\kappa^{'})$, we can arrive at the result $O(\rho^{'}) = O(\kappa^{'}) \sim e^{-2\ell}$ by the same method above. If we assume $\rho^{'} = \alpha e^{-2\ell}$ and $\kappa^{'} = \beta e^{-2\ell}$. Solving α and β, we have $\alpha = \beta = \pm\frac{2}{\sqrt{34}}$ or $\alpha = -\beta = \pm\sqrt{\frac{1}{8}}$.

Substituting the limit value of all the coupling constant and stiffness in the normal phase fixed point into the matrix M, we have:

$$M = \begin{pmatrix} -2 & 0 & 0 & 0 & 0 & 0 \\ 0 & -2 & 0 & 0 & 0 & 0 \\ 0 & 0 & -2 & 0 & 0 & 0 \\ 0 & 0 & 0 & -\infty & 0 & 0 \\ 32\alpha^3 & 0 & \alpha^2(\alpha+\beta) & 0 & -\frac{99\alpha^2+2\alpha\beta}{2} & -\frac{\alpha^2}{2} \\ 0 & 32\beta^3 & \beta^2(\alpha+\beta) & 0 & -\frac{\beta^2}{2} & -\frac{99\beta^2+2\alpha\beta}{2} \end{pmatrix} \tag{S32}$$

Obviously, all the eigenvalues of M are negative which means that the normal phase is a stable fixed point.

(ii)**The charge $4e$ SC phase**:
By the same method above, we rewrite the RG flow equation as following to simplify the calculation:

$$\begin{aligned} \frac{dg_{2,0}}{d\ell} &= (2-\pi\rho^{'})g_{2,0} \\ \frac{d\bar{g}_{0,2}}{d\ell} &= -(2-\pi\kappa^{'})\bar{g}_{0,2} \\ \frac{dg_{1,1}}{d\ell} &= \left(2-\frac{\pi}{4}(\rho^{'}+\kappa^{'})\right)g_{1,1} \\ \frac{dg_4}{d\ell} &= (2-\frac{4}{\pi\kappa^{'}})g_4 \\ \frac{d\rho^{'}}{d\ell} &= -16g_{2,0}^2\rho^{'3} - \frac{1}{2}g_{1,1}^2\rho^{'2}(\rho^{'}+\kappa^{'}) \\ \frac{d\kappa^{'}}{d\ell} &= \frac{256g_4^2}{\pi^4\kappa^{'}} - 16\bar{g}_{0,2}^{-2}\kappa^{'3} - \frac{1}{2}g_{1,1}^2\kappa^{'2}(\rho^{'}+\kappa^{'}), \end{aligned} \tag{S33}$$

We analyze the order of the coupling parameters. Firstly: $O(g_4) \sim e^{-\infty\ell}$, $O(g_{2,0}) < O(e^{-6\ell})$, $O(g_{0,2}) \sim e^{2\ell}$, and $O(g_{1,1}) < O(e^{0\ell})$. we keep the highest order term in the RG flow equation of $\kappa^{'}$:

$$\frac{d\kappa^{'}}{d\ell} = -16g_{0,2}^2\kappa^{'3} \tag{S34}$$

We obtain that $\kappa^{'} \sim e^{-2\ell}$. The differential matrix can be written as:

$$M = \begin{pmatrix} 2-\pi\rho^{'} & 0 & 0 & 0 & -\pi g_{2,0} & 0 \\ 0 & -(2-\pi\kappa^{'}) & 0 & 0 & 0 & \pi\bar{g}_{0,2} \\ 0 & 0 & 2-\frac{\pi}{4}(\rho^{'}+\kappa^{'}) & 0 & -\frac{\pi}{4}g_{1,1} & -\frac{\pi}{4}g_{1,1} \\ 0 & 0 & 0 & 2-\frac{4}{\pi\kappa^{'}} & 0 & \frac{4g_4}{\pi\kappa^{'2}} \\ -32\rho^{'3}g_{2,0} & 0 & -g_{1,1}\rho^{'2}(\rho^{'}+\kappa^{'}) & 0 & -48\rho^{'2}g_{2,0}^2 - \frac{g_{1,1}^2(3\rho^{'2}+2\rho^{'}\kappa^{'})}{2} & -\frac{g_{1,1}^2\rho^{'2}}{2} \\ 0 & \frac{32\kappa^{'3}}{\bar{g}_{0,2}^3} & -g_{1,1}\kappa^{'2}(\rho^{'}+\kappa^{'}) & \frac{512g_4}{\pi^4\kappa^{'}} & -\frac{g_{1,1}^2\kappa^{'2}}{2} & -\frac{256g_4^2}{\pi\kappa^{'2}} - \frac{48\kappa^{'2}}{\bar{g}_{0,2}^2} - \frac{g_{1,1}^2(2\kappa^{'}\rho^{'}+3\kappa^{'2})}{2} \end{pmatrix} \tag{S35}$$

Substituting all the orders of couplings and stiffness into M matrix, we have:

$$M = \begin{pmatrix} <-6 & 0 & 0 & 0 & 0 & 0 \\ 0 & -2 & 0 & 0 & 0 & 0 \\ 0 & 0 & <0 & 0 & 0 & 0 \\ 0 & 0 & 0 & -\infty & 0 & 0 \\ 0 & 0 & 0 & 0 & 0 & 0 \\ 0 & O(e^{0\ell}) & 0 & 0 & 0 & -O(e^{0\ell}) \end{pmatrix} \tag{S36}$$

As we can see, all the eigenvalues are negative except the fifth one which is zero. Obviously, the charge $4e$ SC is a stable phase.

(iii)**The chiral SC phase**:
At the beginning, we rewrite the form of the RG flow equation:

$$\begin{aligned} \frac{dg_{2,0}}{d\ell} &= (2-\pi\rho^{'})g_{2,0} \\ \frac{dg_{0,2}}{d\ell} &= (2-\pi\kappa^{'})g_{0,2} \\ \frac{dg_{1,1}}{d\ell} &= \left(2-\frac{\pi}{4}(\rho^{'}+\kappa^{'})\right)g_{1,1} \\ \frac{d\bar{g}_4}{d\ell} &= -(2-\frac{4}{\pi\kappa^{'}})\bar{g}_4 \\ \frac{d\rho^{'}}{d\ell} &= -16g_{2,0}^2\rho^{'3} - \frac{1}{2}g_{1,1}^2\rho^{'2}(\rho^{'}+\kappa^{'}) \\ \frac{d\kappa^{'}}{d\ell} &= \frac{256}{\pi^4\kappa^{'}\bar{g}_4^2} - 16g_{0,2}^2\kappa^{'3} - \frac{1}{2}g_{1,1}^2\kappa^{'2}(\rho^{'}+\kappa^{'}), \end{aligned} \tag{S37}$$

We analyze the order of all the coupling constants and the stiffness parameters. $O(g_{2,0}) < e^{0\ell}$, $O(g_{0,2}) \sim e^{-\infty\ell}$, $O(g_{1,1}) \sim e^{-\infty\ell}$, and $O(g_4) \sim e^{2\ell}$. We don't need analyze the order of $\rho^{'}$, since it is a fixed parameter in the region $> \frac{2}{\pi}$. And in the last RG flow equation, g_4^2 has the highest order obviously. So, we can neglect other terms in that equation and solve the differential equation to get the order of $\kappa^{'}$. $\kappa^{'} \sim e^{2\ell}$ is obtained.

The differential matrix can be written as:

$$M = \begin{pmatrix} 2-\pi\rho^{'} & 0 & 0 & 0 & -\pi g_{2,0} & 0 \\ 0 & (2-\pi\kappa^{'}) & 0 & 0 & 0 & -\pi g_{0,2} \\ 0 & 0 & 2-\frac{\pi}{4}(\rho^{'}+\kappa^{'}) & 0 & -\frac{\pi}{4}g_{1,1} & -\frac{\pi}{4}g_{1,1} \\ 0 & 0 & 0 & -(2-\frac{4}{\pi\kappa^{'}}) & 0 & -\frac{4\bar{g}_4}{\pi\kappa^{'2}} \\ -32\rho^{'3}g_{2,0} & 0 & -\frac{\rho^{'2}(\rho^{'}+\kappa^{'})}{\bar{g}_{1,1}} & 0 & -48\rho^{'2}g_{2,0}^2 - \frac{g_{1,1}^2(3\rho^{'2}+2\rho^{'}\kappa^{'})}{2} & -\frac{g_{1,1}^2\rho^{'2}}{2} \\ 0 & -32\kappa^{'3}g_{0,2} & -\frac{\kappa^{'2}(\rho^{'}+\kappa^{'})}{\bar{g}_{1,1}} & -\frac{512}{\pi^4\kappa^{'}\bar{g}_4^3} & -\frac{g_{1,1}^2\kappa^{'2}}{2} & -\frac{256g_4^2}{\pi\kappa^{'2}} - \frac{48\kappa^{'2}}{\bar{g}_{0,2}^2} - \frac{g_{1,1}^2(2\kappa^{'}\rho^{'}+3\kappa^{'2})}{2} \end{pmatrix} \tag{S38}$$

Substituting all the limit values of the coupling constant and stiffness parameters at the fixed point, we have:

$$M = \begin{pmatrix} <0 & 0 & 0 & 0 & 0 & 0 \\ 0 & -\infty & 0 & 0 & 0 & 0 \\ 0 & 0 & -\infty & 0 & 0 & 0 \\ 0 & 0 & 0 & -2 & 0 & 0 \\ 0 & 0 & 0 & 0 & 0 & 0 \\ 0 & 0 & 0 & -\infty & 0 & -O(e^{0\ell}) \end{pmatrix} \tag{S39}$$

As we can see, all the eigenvalues are negative except the fifth one which is zero. Obviously, the chiral SC is a stable phase.

(iv)**The chiral metal phase**:

We rewrite the RG flow equation:

$$\begin{aligned}
\frac{d\bar{g}_{2,0}}{d\ell} &= -(2-\pi\rho^{'})\bar{g}_{2,0} \\
\frac{dg_{0,2}}{d\ell} &= (2-\pi\kappa^{'})g_{0,2} \\
\frac{dg_{1,1}}{d\ell} &= \left(2-\frac{\pi}{4}(\rho^{'}+\kappa^{'})\right)g_{1,1} \\
\frac{d\bar{g}_{4}}{d\ell} &= -(2-\frac{4}{\pi\kappa^{'}})\bar{g}_{4} \\
\frac{d\rho^{'}}{d\ell} &= -16\bar{g}_{2,0}^{-2}\rho^{'3}-\frac{g_{1,1}^{2}}{2}\rho^{'2}(\rho^{'}+\kappa^{'}) \\
\frac{d\kappa^{'}}{d\ell} &= \frac{256\bar{g}_{4}^{-2}}{\pi^{4}\kappa^{'}}-16g_{0,2}^{2}\kappa^{'3}-\frac{g_{1,1}^{2}}{2}\kappa^{'2}(\rho^{'}+\kappa^{'}),
\end{aligned} \tag{S40}$$

The order of each constant can be analyzed by the same method as before. $g_{2,0}\sim e^{2\ell}$, $g_{0,2}\sim e^{-\infty\ell}$, $g_{1,1}\sim e^{-\infty\ell}$, $g_{4}\sim e^{2\ell}$, and the RG flow equation of stiffness can be simplified as:

$$\begin{aligned}
\frac{d\rho^{'}}{d\ell} &= -\frac{16\rho^{'3}}{\bar{g}_{2,0}^{2}}; \\
\frac{d\kappa^{'}}{d\ell} &= \frac{256}{\pi^{4}\kappa^{'}\bar{g}_{4}^{2}}
\end{aligned} \tag{S41}$$

Solving the differential equation above, we have $\rho^{'}\sim e^{-2\ell}$ and $\kappa^{'}\sim e^{2\ell}$. At the same time, we can write out the M matrix as following:

$$M=\begin{pmatrix}
-(2-\pi\rho^{'}) & 0 & 0 & 0 & \pi\bar{g}_{2,0} & 0 \\
0 & 2-\pi\kappa^{'} & 0 & 0 & 0 & -\pi g_{0,2} \\
0 & 0 & 2-\frac{\pi}{4}(\rho^{'}+\kappa^{'}) & 0 & -\frac{\pi}{4}g_{1,1} & -\frac{\pi}{4}g_{1,1} \\
0 & 0 & 0 & -(2-\frac{4}{\pi\kappa^{'}}) & 0 & -\frac{4\bar{g}_{4}}{\pi\kappa^{'2}} \\
\frac{32\rho^{'3}}{\bar{g}_{2,0}^{3}} & 0 & -\frac{\rho^{'2}(\rho^{'}+\kappa^{'})}{\bar{g}_{1,1}} & 0 & -\frac{48\rho^{'2}}{\bar{g}_{2,0}^{2}}-\frac{g_{1,1}^{2}(3\rho^{'3}+2\rho^{'}\kappa^{'})}{2} & -\frac{g_{1,1}^{2}\rho^{'2}}{2} \\
0 & -32\kappa^{'3}g_{0,2} & -\frac{\kappa^{'2}(\rho^{'}+\kappa^{'})}{\bar{g}_{1,1}} & -\frac{512}{\pi^{4}\kappa^{'}\bar{g}_{4}^{3}} & -\frac{g_{1,1}^{2}\kappa^{'2}}{2} & -\frac{256\bar{g}_{4}^{-2}}{\pi^{4}\kappa^{'2}}-48\kappa^{'2}g_{0,2}^{2}-\frac{(2\kappa^{'}\rho^{'}+3\kappa^{'2})}{2\bar{g}_{1,1}^{2}}
\end{pmatrix} \tag{S42}$$

Substituting all the values of the coupling constant and stiffness parameters in the fixed point, we arrive at:

$$M=\begin{pmatrix}
-2 & 0 & 0 & 0 & 0 & 0 \\
0 & -\infty & 0 & 0 & 0 & 0 \\
0 & 0 & -\infty & 0 & 0 & 0 \\
0 & 0 & 0 & -2 & 0 & 0 \\
O(e^{0\ell}) & 0 & 0 & 0 & -O(e^{0\ell}) & 0 \\
0 & 0 & 0 & -\infty & 0 & -O(e^{0\ell})
\end{pmatrix} \tag{S43}$$

All the eigenvalues are negative. Obviously, the chiral metal is also a stable phase.

IV. MORE DETAILED RESULTS ABOUT THE RG STUDY

To compare with the phase diagrams with different initial value of the coupling parameters, we present Fig.(S1) in this section. As shown in this figure, we find the direct transition regime between chiral TSF and normal phase are

enhanced with larger initial value of half-vortices couplings $g_{1,1}$. Additionally, chiral metal phase will be enlarged in the phase diagram if we increase the initial value of the coupling parameter g_4.

Our RG results indicate that the interesting phases of charge 4e SC and chiral metal can always exist with different initial coupling parameters.

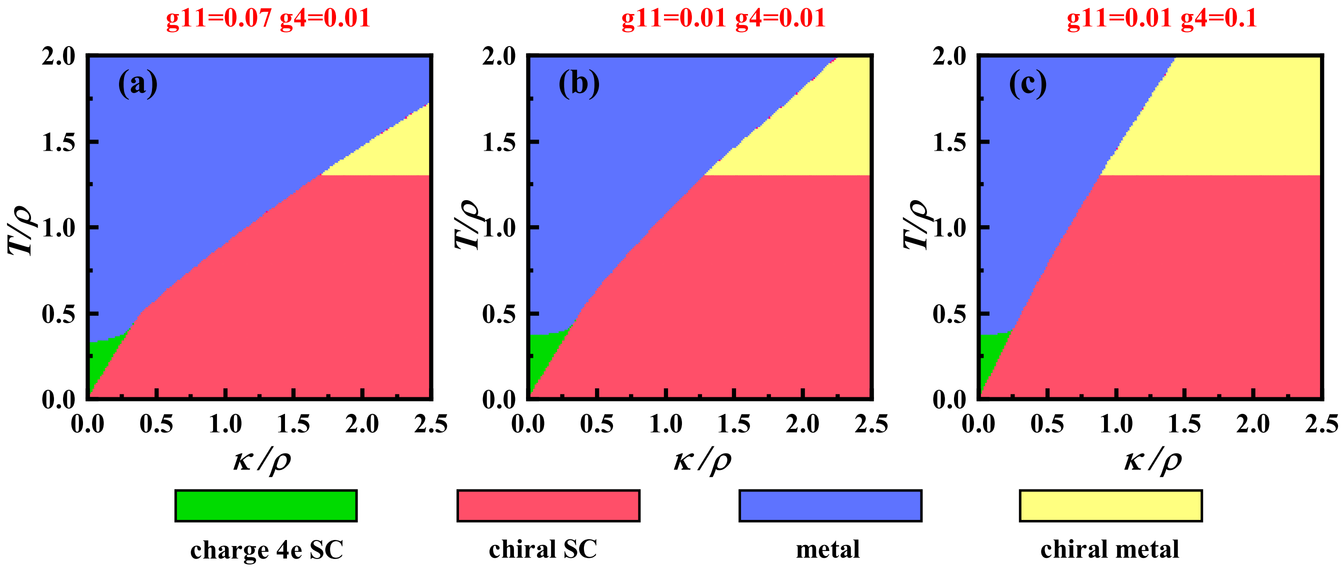

Figure S1. (Color online) Phase diagram provided by the RG approach with different initial coupling parameters. The initial values of the coupling parameters are $g_{2,0} = g_{0,2} = 0.1$, $g_{1,1} = 0.07$ and $g_4 = 0.01$ for (a), $g_{2,0} = g_{0,2} = 0.1$, $g_{1,1} = g_4 = 0.01$ for (b), and $g_{2,0} = g_{0,2} = 0.1$, $g_{1,1} = 0.01$, $g_4 = 0.1$ for (c).

V. MORE DETAILS RESULTS ABOUT THE MC STUDY

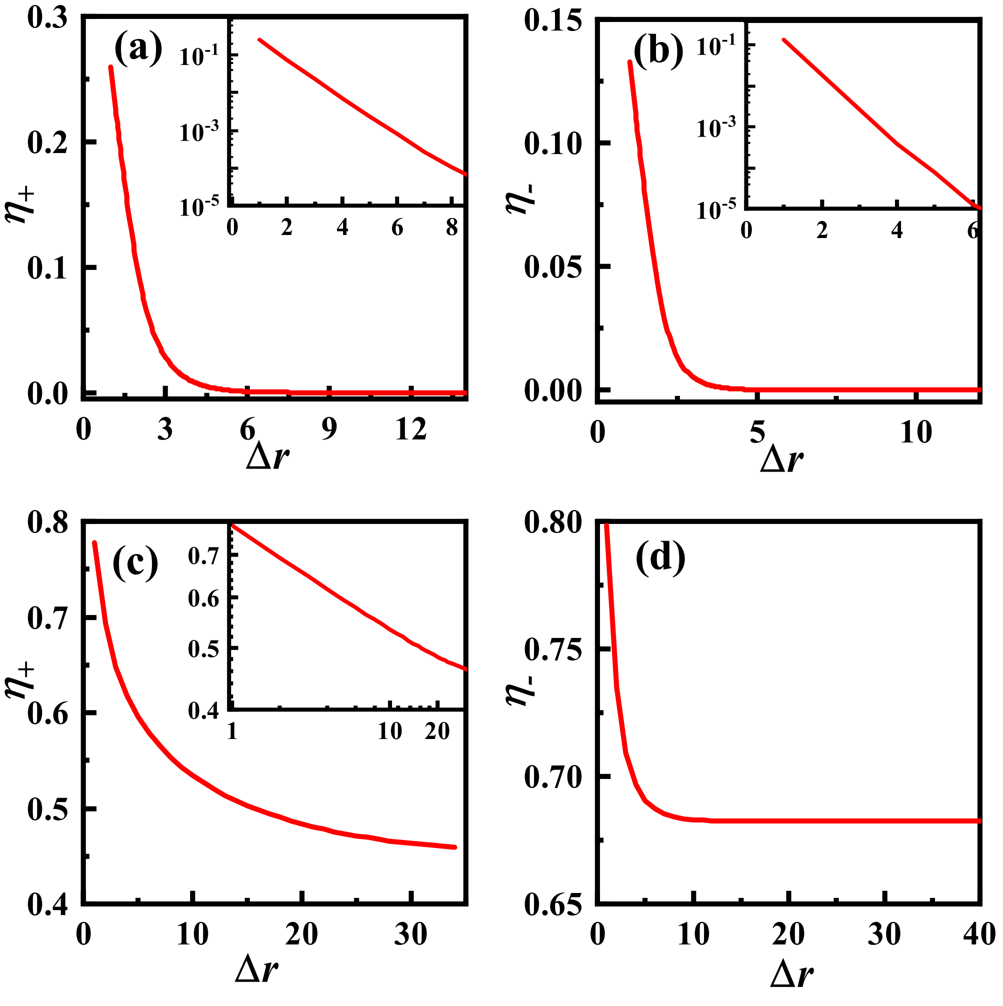

Figure S2. (Color online) (a-b) The correlation function $\eta_\pm$ for the parameter point B($\kappa/\rho = 0.6, T/\rho = 0.45$) in Fig.2(b) in the main text, respectively. The y- axes of the inset are logarithmic axes. (c) The correlation function η_+ for the parameter point C($\kappa/\rho = 1, T/\rho = 0.2$) in Fig.2(b) in the main text, both the x- and y- axes of the inset are logarithmic axes. (d) The correlation function η_- for the parameter point C in Fig.2(b) in the main text, the y- axis of the inset is logarithmic axis.

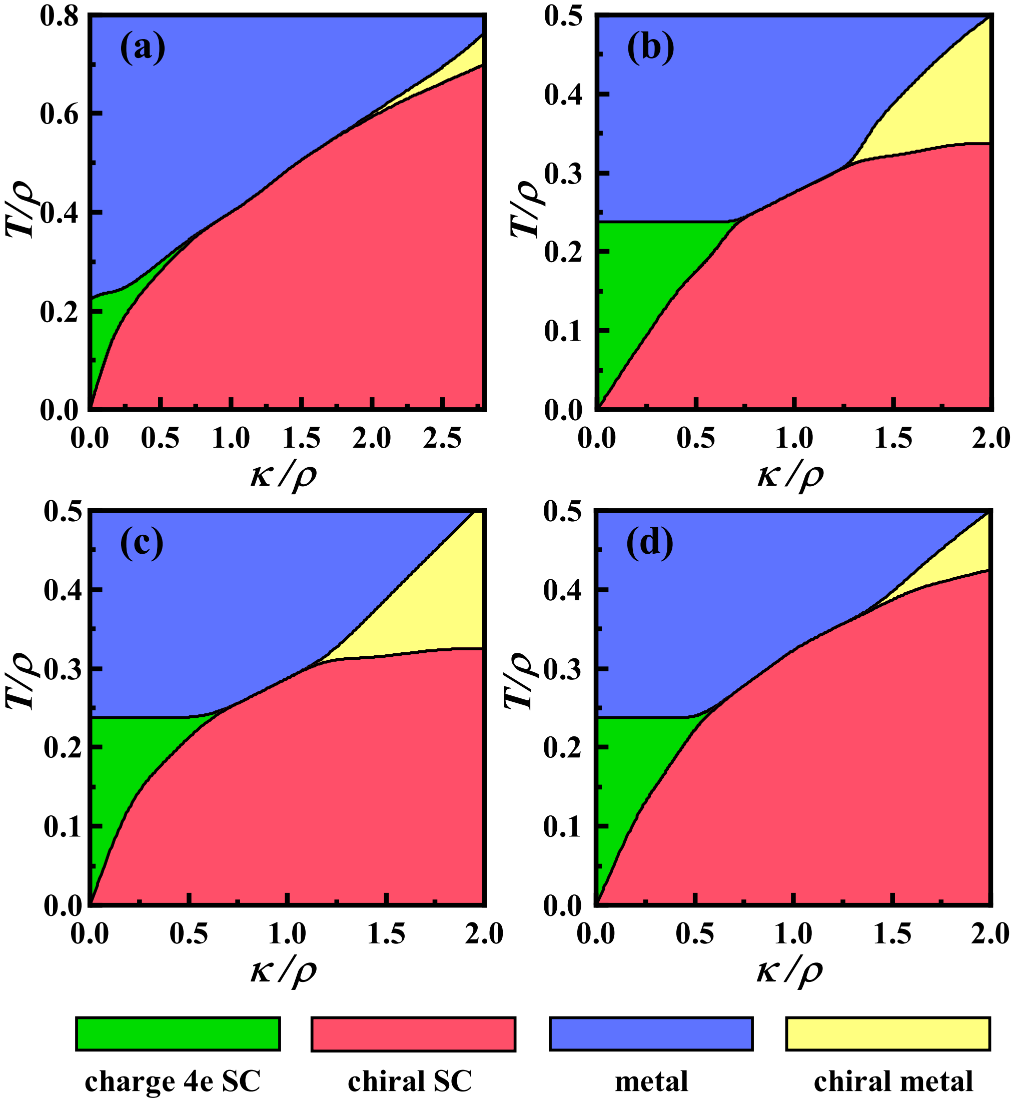

Figure S3. (Color online) Phase diagram provided by the MC study with different parameter γ and extra B term. (a) The same parameters as those in the phase diagram in the main text except that $\gamma = \rho\kappa/2(\rho+\kappa)$. (b) The same parameters as those in the phase diagram in the main text except that $\gamma = \rho\kappa/6(\rho+\kappa)$. (c) The same parameters as those in the phase diagram in the main text except that $\gamma = 0.1\rho$ is a constant. (d)The same parameters as those in the phase diagram in the main text except that a weak first-order Josephson coupling with coefficient $B = 0.01\rho$ is added.

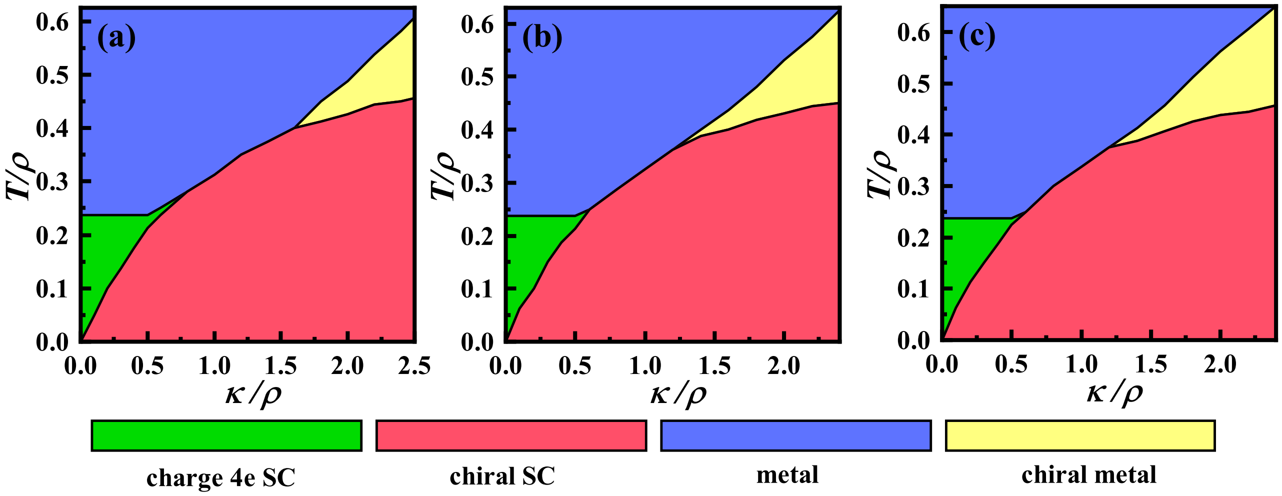

Figure S4. (Color online) Phase diagram provided by the MC study with different parameter A. (a-c) The same parameters as those in the phase diagram in the main text except that $A = 0.0125\rho, 0.05\rho$ and 0.1ρ, respectively.

The properties of the correlation function of the parameter point B and C in phase diagram is shown in Fig. S2. For the parameter point B, Fig. S2(a) and (b) show the correlation functions η_+ and η_-, respectively. Both the correlation function η_+ and η_- are exponentially decay, which proves that point parameter B is the metal state. On the contrary, For the parameter point C, Fig. S2(c) and (d) show the correlation functions η_+ and η_-, respectively. The correlation function η_+ is power law decay but the correlation function η_- is a constant, which proves that parameter point D is the chiral SC.

To verify the generality of the discretized Hamiltonian, we perform the MC study with different γ and A to obtain the phase diagram, shown in Fig. S3 (a-c) and Fig. S4 (a-c). The phase diagrams for different γ do not change qualitatively.

To verify the stability of the results, we perform the MC study with a weak first-order Josephson-coupling term added, whose coefficient is $B = 0.01\rho$. The γ is the same as that adopted in the main text. The phase diagram is shown in Fig. S3(d), which is similar with that obtained for zero B.

VI. THE MC RESULT WITHOUT CONSIDERING KINEMATIC CONSTRAINT($\gamma = 0$).

In order to highlight the importance of γ term in the Eq. (15) in main text, we calculate the phase diagram with $\gamma = 0$ and present the theoretical explanation about this phase diagram.

If we turn off the γ term in Eq. (15) in main text, we have $\alpha = \rho/4, \lambda = \kappa/4$. Then we redo the Monte-Carlo calculations. Consequently, the obtained phase diagram is displayed in the following Fig. S5 (a). This phase diagram is very simple, which is divided by two lines into four phases touching at one qua-critical point. The straight line parallel to the x-axis represents the K-T transition, suggesting that the (quasi-) ordering temperature of θ_+ only relies on ρ. The line passing through the coordinate origin represents the Ising transition, suggesting that the ordering temperature of θ_- only relies on κ when fixing A. This phase diagram suggests that θ_+ and θ_- are decoupled, which is analytically understood as follow.

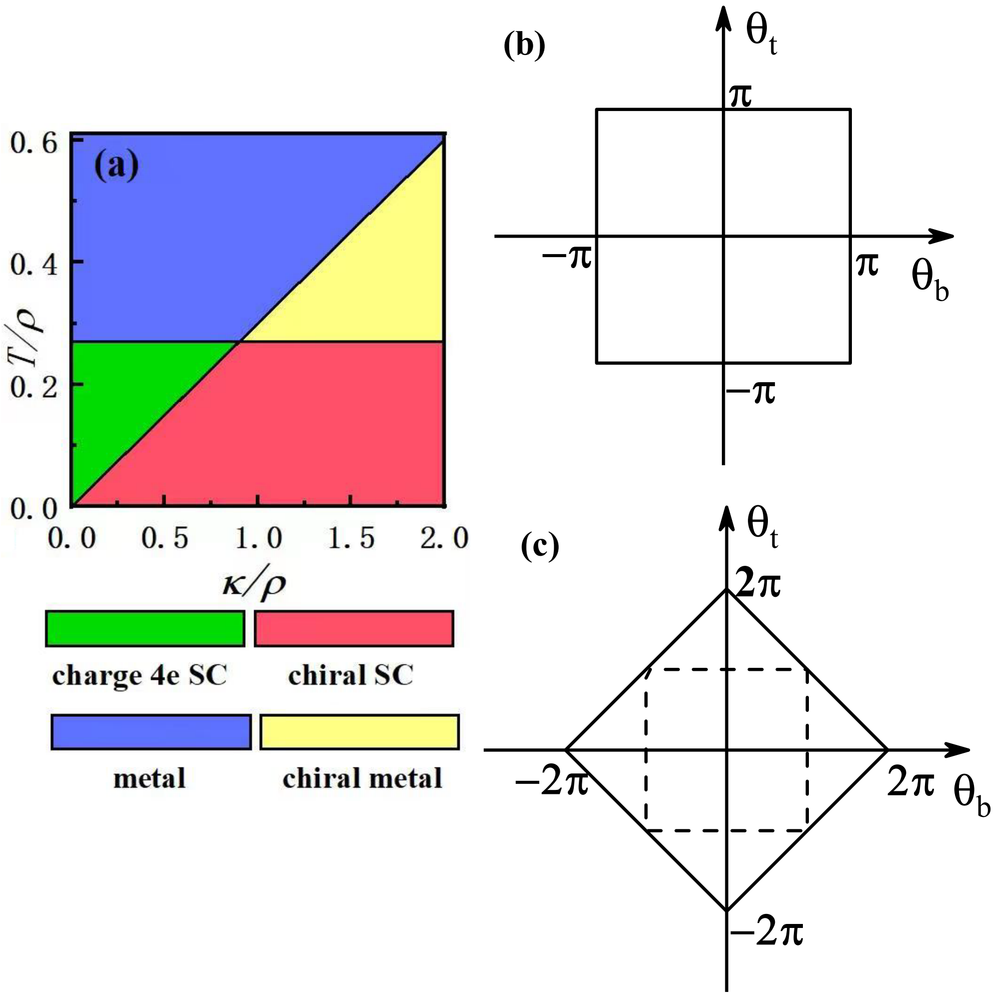

Figure S5. (Color online) (a) Phase diagram provided by the MC study with different parameter γ. The same parameters as those in the phase diagram in the main text except that $\gamma = 0$. (b) Integral region of θ_b and θ_t at a given site. (c) Expanded integral region of θ_b and θ_t at a given site.

The partition function of the model is written as

$$Z = \int \dots \int \prod_{\vec{r}_i} d\theta_t(\vec{r}_i) d\theta_b(\vec{r}_i) e^{-\beta H[\{\theta_t(\vec{r}_i), \theta_b(\vec{r}_i)\}]} \tag{S44}$$

For each site, the integral region is within the "first Brilloiun Zone (BZ)" $\theta_t \in [-\pi, \pi)$, $\theta_b \in [-\pi, \pi)$ shown in Fig. S5 (b). Since $H[\{\theta_t(\vec{r}_i), \theta_b(\vec{r}_i)\}]$ is a periodic function of θ_b and θ_t with period 2π, the integral region can be expanded to the "second BZ" shown in Fig. S5 (c), i.e. $\theta_t + \theta_b \in [-2\pi, 2\pi)$, $\theta_t - \theta_b \in [-2\pi, 2\pi)$ or equivalently $\theta_+ \in [-\pi, \pi)$, $\theta_- \in [-\pi, \pi)$. Such an expansion only doubles Z, and would not change the physics. For $\gamma = 0$, we have $H = H_+[\{\theta_+(\vec{r})\}] + H_-[\{\theta_-(\vec{r})\}]$, and then we have

$$\begin{aligned} Z &= \int \cdots \int \prod_{\vec{r}_i} d\theta_+(\vec{r}_i) d\theta_-(\vec{r}_i) e^{-\beta H_+[\{\theta_+(\vec{r}_i)\}]} \cdot e^{-\beta H_-[\{\theta_-(\vec{r}_i)\}]} \\ &= \int \dots \int \prod_{\vec{r}_i} d\theta_+(\vec{r}_i) e^{-\beta H_+[\{\theta_+(\vec{r}_i)\}]} \cdot \int \dots \int \Pi_{\vec{r}_i} d\theta_-(\vec{r}_i) e^{-\beta H_-[\{\theta_-(\vec{r}_i)\}]} \\ &= Z_+ \cdot Z_- \end{aligned} \tag{S45}$$

This result explains why θ_+ and θ_- are decoupled for $\gamma = 0$.

However, since we do not consider kinematically correlated of θ_+ and θ_-, the phase diagram shown in the Fig. S5 (a) is topologically different from Fig. 2 in the main text and is wrong.

[1] Meng Zeng, Lun-Hui Hu, Hong-Ye Hu, Yi-Zhuang You, and Congjun Wu, arXiv: 2102.06158.
